# Silencing NIK potentiates anti-VEGF therapy in a novel 3D model of colorectal cancer angiogenesis

**DOI:** 10.18632/oncotarget.25442

**Published:** 2018-06-19

**Authors:** Chrissta X. Maracle, Kim C.M. Jeucken, Boy Helder, Thomas M. van Gulik, Anne Steins, Hanneke W.M. van Laarhoven, Sander W. Tas

**Affiliations:** ^1^ Amsterdam Rheumatology and Immunology Center, Academic Medical Center/University of Amsterdam, Amsterdam, The Netherlands; ^2^ Laboratory for Experimental Immunology, Academic Medical Center/University of Amsterdam, Amsterdam, The Netherlands; ^3^ Department of Surgery, Academic Medical Center/University of Amsterdam, Amsterdam, The Netherlands; ^4^ Department of Medical Oncology, Academic Medical Center/University of Amsterdam, Amsterdam, The Netherlands

**Keywords:** tumor angiogenesis, NIK, bevacizumab, colorectal cancer, cytokines

## Abstract

Angiogenesis is essential for colorectal cancer (CRC) progression, as demonstrated by the beneficial clinical effects of therapeutics inhibiting VEGF signaling. However, alternative mechanisms of neovascularization can develop, resulting in treatment failure. Previously we demonstrated NF-κB-inducing kinase (NIK) contributes to pathological angiogenesis. Here, we investigate NIK as a therapeutic target in endothelial cells (EC) in CRC. To determine NIK expression levels in CRC tissues, we immunostained both primary colorectal tumors and tumors metastasized to the liver. Additionally, a 3D tumor-stromal cell interaction model was developed including EC, fibroblasts and CRC cells to study tumor angiogenesis. This model tested efficacy of NIK-targeting siRNA (siNIK) in EC alone or in combination with the anti-VEGF antibody, bevacizumab. Both primary CRC and liver metastases contained blood vessels expressing NIK. In patients receiving chemotherapy plus bevacizumab, immature NIK^+^ vessels (*p* < 0.05) were increased as compared to chemotherapy alone. Activation of NIK by lymphotoxin-beta receptor (LTβR) induced increases in pro-angiogenic mediators, including interleukin (IL)-6, IL-8, chemokine (C-X-C motif) ligand (CXCL)1 and CXCL5 in EC and fibroblasts, accompanied by sprouting in the 3D model, which was blocked by siNIK in EC. Treatment with bevacizumab plus siNIK in EC resulted in a synergistic effect and reduced VEGF and bFGF-induced sprouting (*p* < 0.05). Here, we demonstrate a role for NIK in CRC-associated angiogenesis. Targeting NIK in EC in combination with anti-VEGF antibody bevacizumab may hold therapeutic potential to increase efficiency in blocking tumor neovascularization, either to prevent treatment failure due to activation of accessory pathways such as NF-κB signaling or as a rescue treatment.

## INTRODUCTION

Over the past decades, colorectal cancer (CRC) incidence has steadily risen and is currently the third most common type of such malignancy, accounting for approximately 10% of cancer related deaths in Western countries [1, 2]. A crucial component of cancer progression is angiogenesis, which is defined as the formation of new blood vessels from pre-existing vasculature, as an increase in oxygen and nutrient availability is needed for sustained tumor growth to occur [3]. Moreover, angiogenesis is also thought to facilitate metastasis formation, although its influence in this capacity can vary between cancers [4]. In CRC, metastasis and angiogenesis are strongly linked, with several studies demonstrating that targeting molecules which enhance angiogenesis can also affect metastasis [5–7].

Vascular endothelial growth factor (VEGF) is a key cytokine in blood vessel formation and is a therapeutic target of the monoclonal antibody bevacizumab, currently one of the most widely used inhibitors of angiogenesis. In the context of colorectal cancer, it is considered a first-line treatment as it has been demonstrated to increase overall survival when combined with various regimens of chemotherapy [8]. However, refractoriness to treatment often occurs, and neovascularization resumes, which then allows for tumor growth to proceed [9, 10]. Resistance to VEGF inhibition generally develops through compensatory mechanisms of angiogenesis, including activation of alternative signaling pathways in endothelial cells (EC) by other growth factors and/or inflammatory molecules. The latter can also activate surrounding stromal and immune cells within the tumor microenvironment, helping to further ignite and enhance tumor neovascularization [11, 12].

Inflammation and pathological angiogenesis are two processes that are closely linked, and the NF-κB family of transcription factors is known to be important to both [13, 14]. NF-κB signaling can be activated via the canonical and the noncanonical pathways. The canonical NF-κB pathway is central to regulating inflammatory responses and can be triggered by ligands such as tumor necrosis factor alpha (TNFa), interleukin (IL)-1, IL-6 and lipopolysaccharide (LPS) [15]. Several studies have demonstrated that this pathway contributes to neovascularization in various models of tumor angiogenesis [16, 17]. The noncanonical pathway is more specific in its function and is essential for adaptive immune responses and the development of lymph nodes [18]. Increasing evidence also suggests the involvement of noncanonical NF-kB signaling in cancer pathogenesis, as many of the activating receptors of this pathway, such as the lymphotoxin-beta receptor (LTβR) and cluster of differentiation (CD) 40, are present in the tumor microenvironment and have been implicated in processes such as tumorigenesis [19–21]. Interestingly, the main regulating kinase of the noncanonical pathway, NF-κB inducing kinase (NIK), was recently found to be upregulated in colorectal cancer tumor tissues and determined to be important for tumor progression [22].

Previously, we demonstrated that NIK is highly expressed in blood vessels of primary colorectal cancer tissues, but not in healthy human colon [23]. Furthermore, activation of the noncanonical NF-kB pathway promotes angiogenesis, suggesting that NIK may be contributing to tumor neovascularization [23]. Here, we expand upon those initial studies and further characterize NIK expression in primary and metastatic CRC tissues. More specifically, we examine tissues from patients being treated with the anti-angiogenic agent bevacizumab, as we hypothesize that NIK-induced noncanonical NF-κB signaling may act as an accessory pathway when classical angiogenic mechanisms are blocked. Furthermore, we describe a novel 3-dimensional (3D) *in vitro* model of CRC to study tumor stromal cell interaction in the context of angiogenesis and ultimately test if combined targeting of NIK and VEGF can have synergistic effects in blocking tumor vascularization.

## RESULTS

### NIK^+^ blood vessels are present in CRC liver metastasis with increased levels of immature NIK^+^ vessels in patients treated with bevacizumab

**Figure 1:**
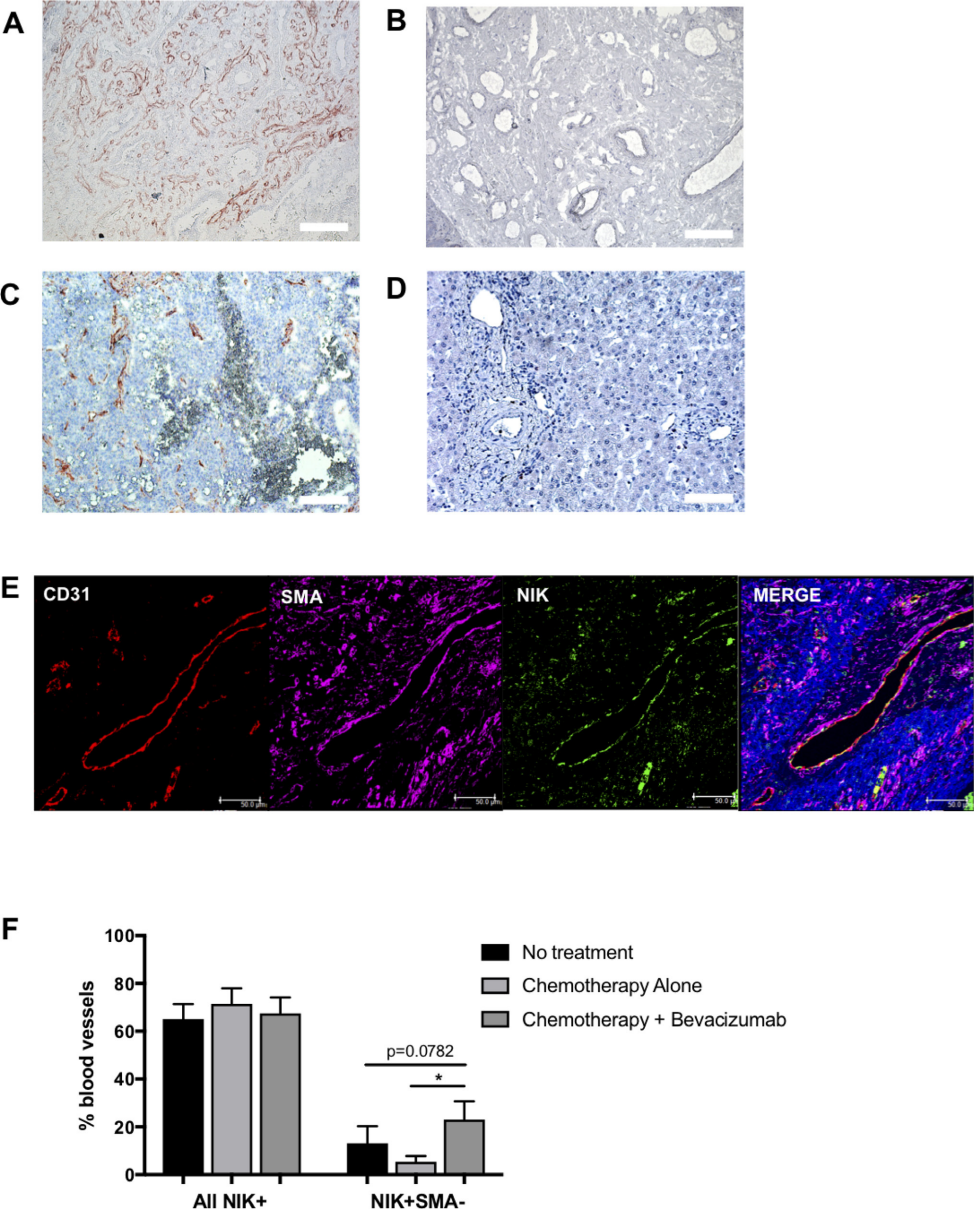
NIK expression in colorectal cancer tissues Immunohistochemical stain of NF-kappaB inducing kinase (NIK) in (**A**) healthy colon, (**B**) colorectal cancer primary tumor, (**C**) healthy liver and (**D**) colorectal cancer associated tumor metastasis of the liver (*n* = 5). (**E**) Immunofluorescent image of metastatic tumor associated vasculature of the liver using the endothelial marker CD31, NIK and smooth muscle cell marker SMA. (**F**) Quantification of NIK positive vessels in metastatic liver tissue of patients undergoing no treatment (*n* = 13), chemotherapy (Chemo only; *n* = 10) or chemotherapy plus Bevacizumab (Chemo +Bev; *n* = 9).

Previously, we reported active noncanonical NF-κB signaling in the blood vessels of various tumors, with the detection of stable NIK expression in the vasculature of tumor tissues but not in healthy tissues [23]. Here, we report that primary CRC biopsies contain a remarkably high level of NIK positive (NIK^+^) blood vessels (Figure 1A) whereas a complete absence of NIK is observed in blood vessels of healthy colon tissue (Figure 1B). To determine if vascular NIK expression was characteristic only of primary CRC tumors or was also present in metastases, we investigated the levels of NIK stabilization in blood vessels of CRC liver metastases. Using colorectal cancer metastases removed during liver surgery, we found that NIK stabilization was also occurring in blood vessels at these sites of secondary tumor growth (Figure 1C), whereas it was not observed in healthy liver (Figure 1D). This indicates that noncanonical NF-κB signaling is occurring within the metastatic tumor vasculature and suggests that activating mechanisms of the pathway are not restricted to primary tumors.

Within our cohort, patients received one of three different treatment regimens before undergoing the resection procedure: 1) no treatment, 2) chemotherapy alone or 3) a combination of chemotherapy and the VEGF-targeting antibody bevacizumab. Immunohistochemical staining for the EC marker CD31, blood vessel maturation marker smooth muscle actin (SMA), and NIK, demonstrated that NIK is expressed in both mature (SMA^+^) and immature (SMA^-^) blood vessels (Figure 1E). The analysis also revealed that although there were no differences in the percentage of total NIK^+^ blood vessels between the 3 patient groups, there was a significant increase (*p* = 0.0417) in the percentage of immature NIK^+^ vessels in patients receiving chemotherapy in combination with bevacizumab as compared to those undergoing chemotherapy alone (Figure 1F). A similar trend was also observed between the bevacizumab group and patients receiving no treatment (*p* = 0.0782). These findings suggest that the blockade of VEGF signaling may result in increased activation of the noncanonical NF-κB pathway in immature blood vessels in metastatic CRC.

### Activation of the noncanonical pathway induces angiogenesis in a novel 3D tumor stroma interaction model

**Figure 2:**
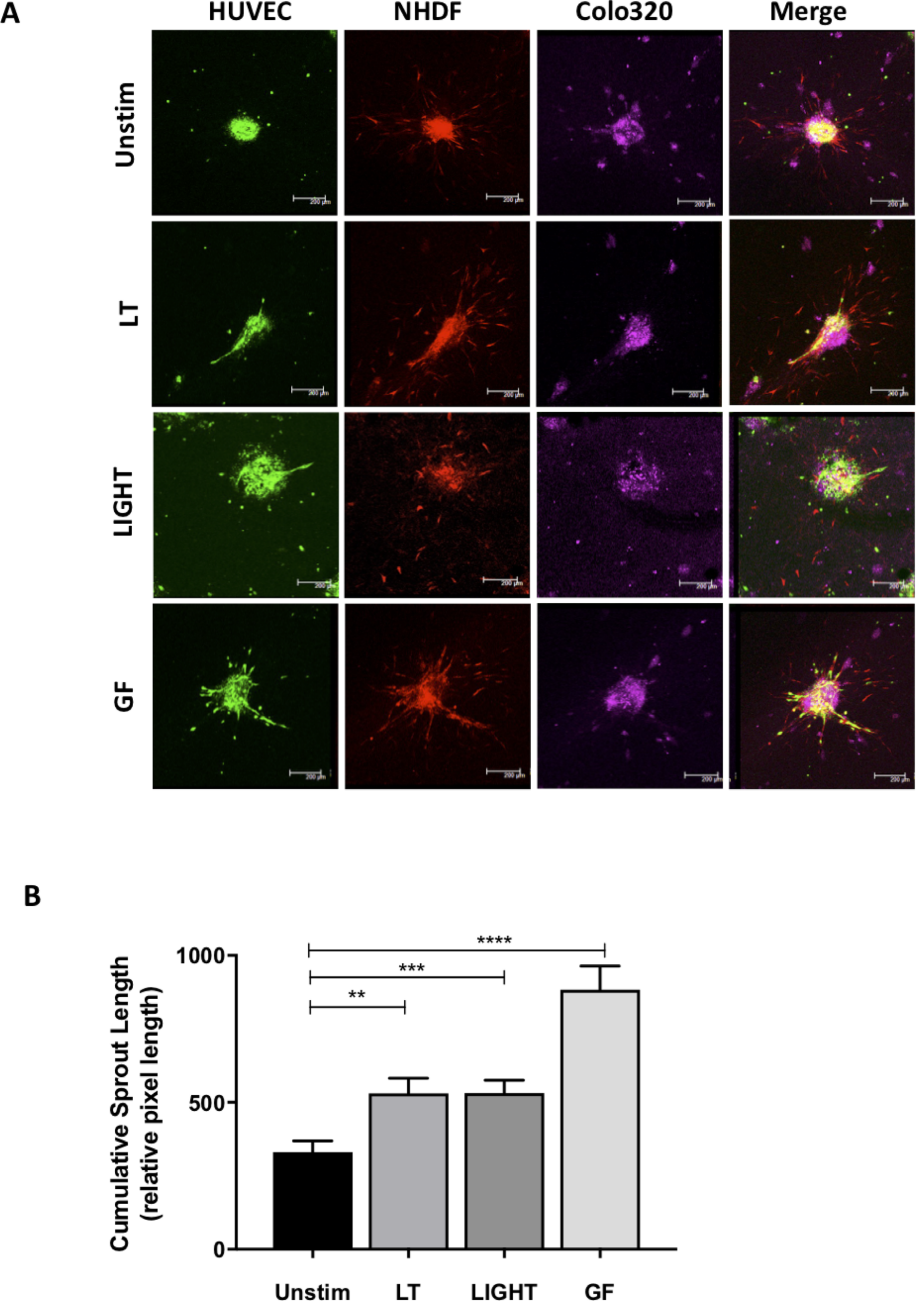
Activation of the non-canonical pathway induces angiogenesis in the 3D tumor-stroma model (**A**) Confocal microscopic images of spheroids containing ECs (HUVEC), fibroblasts (NHDF) and colorectal cancer cells (Colo320) stimulated with activators of the non-canonical pathway lymphotoxin (LT) or LIGHT as compared to controls unstimulated and growth factors VEGF/bFGF (GF). (**B**) Quantification of cumulative sprout length in spheroids unstimulated or stimulated with LT, LIGHT or GF. (*n* = 19–38 spheroids per condition). (^**^represents *p* < 0.01; ^***^represents *p* < 0.001, ^****^represents *p* < 0.0001).

To determine whether activation of the noncanonical NF-κB pathway can indeed promote angiogenesis associated with CRC, we developed a novel 3D tumor stroma interaction model, consisting of CRC cells (Colo320-HRS), EC (HUVEC) and fibroblasts (NHDF), which are combined to make singular tumor spheroids. To visualize the different cell types within the spheroids, we pre-labeled cells using cell tracker dye, tagging the EC green, the fibroblasts red and the CRC cells violet (Figure 2A). Next, we used the cumulative sprout length of vessel like structures formed by EC (green) to compare differences in sprout formation. Interestingly, we also observed that the EC and fibroblasts were often overlapping, particularly in the formation of sprouts, suggesting a support network between the two components of the stromal compartment (Supplementary Figure 1).

In order to activate the noncanonical NF-κB pathway, we stimulated spheroids with ligands of the lymphotoxin beta receptor (LTβR), specifically lymphotoxin (LT) or TNF super family member 14 (TNFSF14), also known as LIGHT, and then compared them to those stimulated with the growth factors (GF), VEGF and basic fibroblast growth factor (bFGF), which acted as classical promoters of angiogenesis. Upon stimulation with LT or LIGHT, we measured significant increases in cumulative sprout length (*p* < 0.01; *p* < 0.001), resulting in a near 2-fold increase (Figure 2B) as compared to untreated control spheroids. Immunofluorescence staining revealed that NIK was activated in the spheroid EC as co-localizaion was observed between NIK and CD31 positive cells (Supplementary Figure 2). These levels of sprouting however, did not reach that of those induced by growth factors (*p* < 0.0001), suggesting that sprouting promoted by the LTβR is not as potent as that of the VEGF and bFGF signaling cascades. Interestingly, the same increase of LTβR-induced sprouting was also observed under hypoxic conditions (Supplementary Figure 3). These results demonstrate that multicellular tumor-like spheroids can be generated and used as a readout for angiogenesis, and more importantly, that activation of the noncanonical NF-κB pathway supports CRC-associated neovascularization.

### LTβR activation in stromal cells leads to transcriptional upregulation of the proangiogenic cytokines IL-6, IL-8, CXCL5 and CXCL1

**Figure 3:**
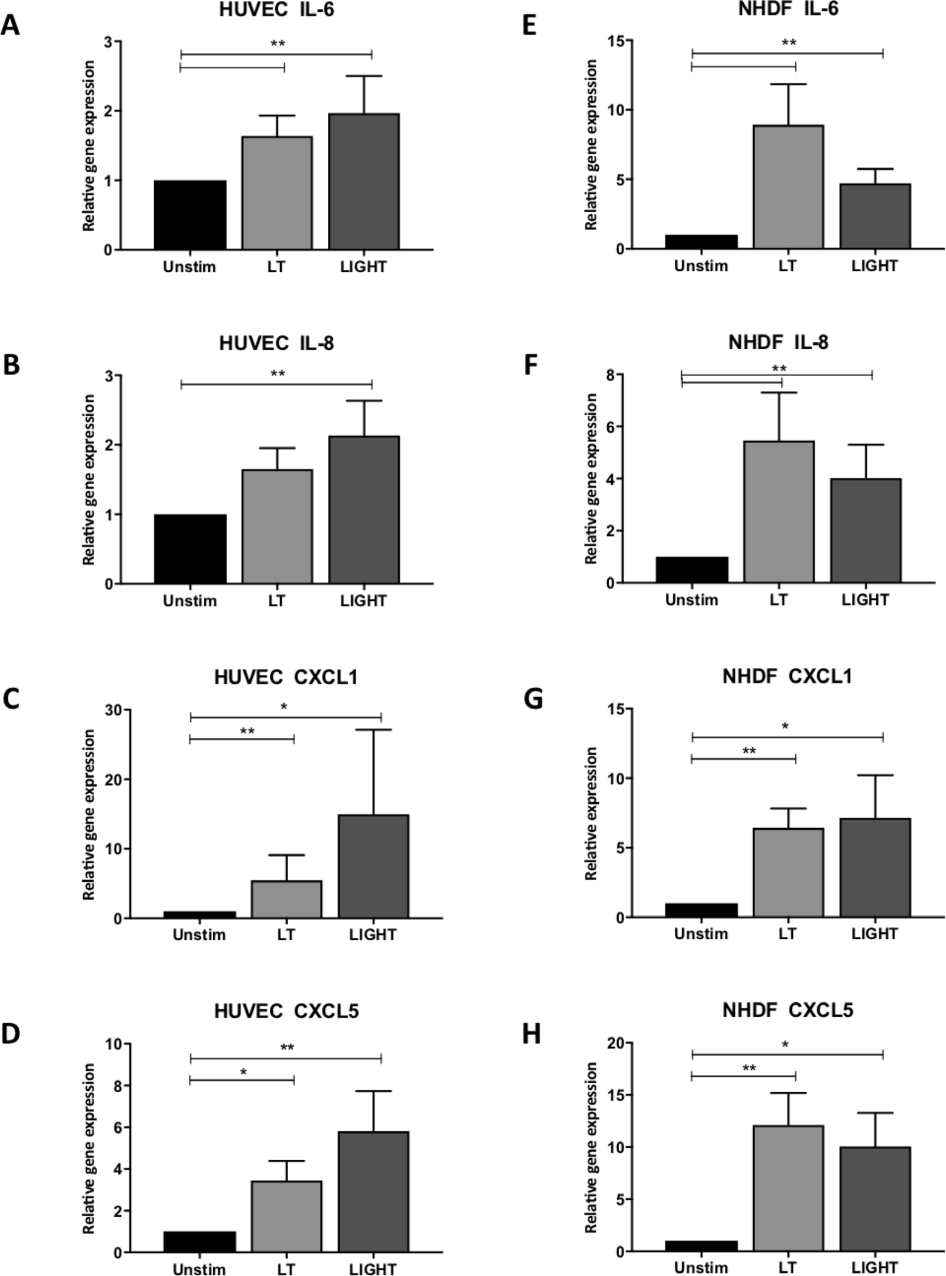
Activation of the non-canonical NF-kappa B pathway induces expression of pro-angiogenic factors in stromal cells HUVEC gene expression of (**A**) interleukin 6 (IL-6) (**B**) IL-8 (**C**) chemokine (C-X-C motif) ligand 1 (CXCL1) and (**D**) CXCL5, upon stimulation with LT or LIGHT, by real-time quantitative polymerase chain reaction (*n* = 6). Similarly, NHDF transcript levels of (**E**) IL-6, (**F**) IL-8, (**G**) CXCL1 and (**H**) CXCL5 (*n* = 6) following activation of the LTβR. (^*^signifies *p* < 0.05; ^**^signifies *p* < 0.01).

To gain insight into possible mechanisms behind the observed sprout formation, we further examined how the individual cell types responded to activation of the noncanonical NF-κB pathway enhancing vessel formation in the 3D model. To investigate this, cell types were cultured individually and transcript levels of VEGF in addition to several cytokines known to be both pro-inflammatory and pro-angiogenic, were measured. VEGF was detected in all three cell types under basal conditions and as expected, was increased in hypoxia (Supplementary Figure 4). LTβR activation did not increase the transcript levels in normoxic conditions, while in hypoxia, VEGF was heightened in fibroblasts stimulated with LIGHT (Supplementary Figure 4). With respect to the inflammatory molecules, only stromal cells had measurable values. In EC, significant increases in IL-6 (*p* < 0.01; *p* < 0.01), IL-8 (*p* < 0.01; *p* < 0.01), chemokine (C-X-C motif) ligand (CXCL) 1 (*p* < 0.01; *p* < 0.05) and CXCL5 (*p* < 0.05; *p* < 0.01) were observed following LTβR activation by LT or LIGHT, respectively (Figure 3A–3D). To confirm activation of the noncanonical pathway, increased transcript levels of NIK following LT and LIGHT stimulation were observed, in addition to enhanced p100 processing to p52, which is indicative of NIK activation (Supplementary Figure 5). Likewise, these transcripts were significantly upregulated in NHDF (Figure 3E–3H). Notably, we again measured similar trends under low oxygen conditions (Supplementary Figure 6) suggesting that the noncanonical pathway is not affected by hypoxia.

### Targeting NIK in ECs abrogates LTβR-induced sprouting and potentiates the angiostatic effects of bevacizumab

**Figure 4:**
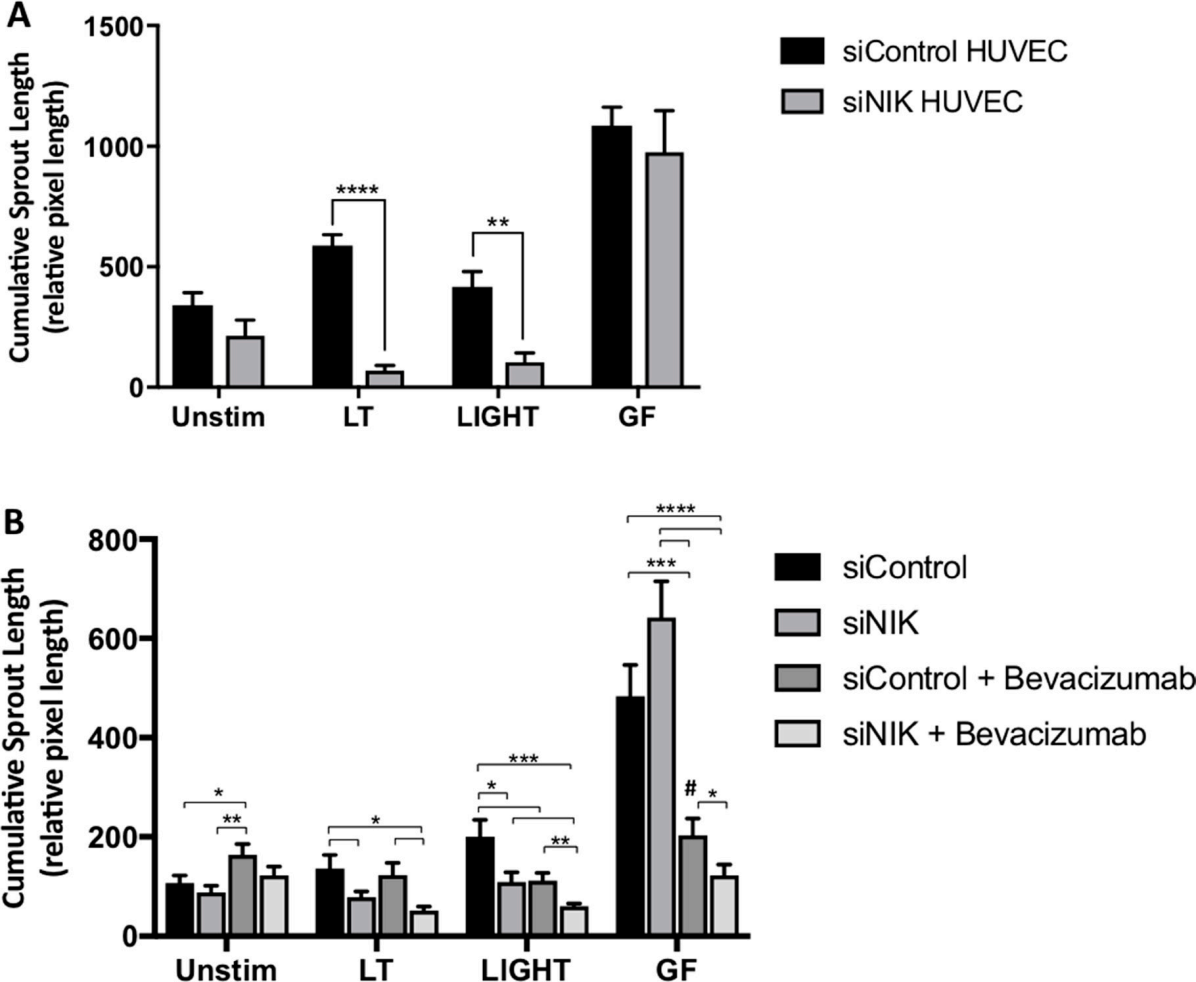
Targeting NIK in HUVEC attenuates angiogenesis in the 3D tumor stroma model (**A**) Cumulative sprout formation of spheroids containing HUVEC pretreated with a non-targeting (siControl) or NIK targeting siRNA (siNIK), combined with non-treated NHDF and Colo320 cells. Spheroids were stimulated with activators of the non-canonical pathway lymphotoxin (LT) and LIGHT or with the growth factors VEGF/bFGF (GF), (*n* = 4–6 spheroids per condition). (**B**) In addition to pretreating cells with either non-targeting or NIK targeting siRNA, the VEGF blocking antibody Bevacizumab (125 µg/mL) was also administered and cumulative sprout formation determined (*n* = 7–12 spheroids per condition). (*signifies *p* < 0.05; ^**^signifies *p* < 0.01, ^***^signifies *p* < 0.001; ^****^signifies *p* < 0.0001; ^#^signifies *p* < 0.001 as compared to unstimulated, siControl).

Following the determination that NIK is stably expressed in blood vessels of both primary CRC and liver metastases, and that NIK-mediated activation of the noncanonical NF-κB pathway induces angiogenesis, we further investigated whether targeting of NIK in ECs alone could abrogate sprouting in the tumor stroma interaction model. To answer this, we pre-treated EC with either NIK targeting (siNIK) or non-targeting siRNA (siControl) before incorporating them into spheroids, followed by stimulation with LT, LIGHT or GF. Importantly, sprout formation promoted through the induction of LTβR-signaling was significantly reduced in the spheroids containing siNIK-treated EC as compared to siControl (Figure 4A). These observations were specific to LT and LIGHT stimulated spheroids, as no significant reduction in cumulative sprout formation was recorded in cells activated by GF, which was anticipated, as growth factor signaling is not directly linked to the noncanonical NF-κB pathway. Taken together, these data demonstrate that LTβR-induced sprouting in CRC spheroids is NIK dependent, whereas GF-induced sprouting is not.

Since our findings suggest that the noncanonical NF-κB pathway is activated in CRC blood vessels and may be acting as an alternative pathway promoting angiogenesis, especially when VEGF signaling is blocked, we postulated that targeting of NIK could impede this secondary mechanism contributing to tumor neovascularization. Using the 3D model, we compared the ability of targeting NIK in ECs versus bevacizumab in blocking angiogenesis, and then further investigated whether there was a possible synergistic effect in the combination of treatments. For this, we compared spheroids containing EC pre-treated with either siControl or siNIK, with or without the addition of bevacizumab (Supplementary Figure 7).

Spheroids stimulated with LT again showed a significant decrease in sprout formation after siNIK-treatment of EC (*p* < 0.05), however, bevacizumab did not demonstrate an additive effect (Figure 4B). Further analysis revealed that cumulative sprout formation induced by LIGHT however, was significantly reduced not only by siNIK but by bevacizumab as well (*p* < 0.05), which was further enhanced when the two treatments were combined (*p* < 0.001). Lastly, the addition of bevacizumab to spheroids stimulated with GF resulted in a stark drop in cumulative sprout formation (*p* < 0.001) as compared to untreated siControl, however the levels of sprouting were still significantly higher than those of unstimulated spheroids with siControl (*p* < 0.001). When siNIK-treatment of EC was combined with bevacizumab, GF-induced vessel formation declined even further, with sprout length reaching similar levels as the basal non-stimulated CRC spheroids (*p* < 0.05), suggesting an additive effect of combining the two treatments. These observations are striking as the synergistic impact of targeting NIK and VEGF has not yet been described.

## DISCUSSION

The current treatment strategies targeting VEGF signaling in CRC through compounds such as bevacizumab have proven their clinical utility by improving progression free survival, as well as overall survival [24]. However, challenges still remain, as durability of the response is limited and resistance to treatment likely. Thus it is critical to understand the mechanisms driving resistance and, importantly, targeting of such secondary processes could improve patient outcomes.

Here we describe the noncanonical NF-κB pathway as a possible alternative mechanism supporting tumor angiogenesis. Stable expression of the signaling cascade’s main regulating kinase, NIK, is found extensively throughout the tumor vasculature both in primary CRC and at secondary sites of liver metastases. This phenomenon is wholly related to pathological tissue, as NIK expression is not found in healthy colon or liver. Importantly, the stabilization and subsequent activation of NIK appears in both mature and immature vessels, but it is the number of immature NIK^+^ vessels that is increased in patients treated with bevacizumab. This may indicate that the noncanonical NF-κB pathway is activated in newly formed vessels and acts as a substitute mechanism for neovascularization when VEGF signaling is blocked. However, as tumor-associated vascular networks are generally deemed messy and immature in nature, it is unclear whether these vessels are truly nascent or if the lack of VEGF acts only as a catalyst for activation of NIK in the existing endothelium.

As tumor angiogenesis is a complex process involving several different cell types, we developed a model that included the key elements of tumor stroma interactions and can be used for studying CRC-related angiogenesis. Utilizing this tool, we have demonstrated that activation of the LTβR, and subsequently the noncanonical NF-κB pathway, induces sprouting of vessel like structures in spheroids containing CRC cells, fibroblasts and ECs. Of relevance, the activating ligands of this receptor, LT and LIGHT, are highly expressed in CRC tissues [25]. Examining the cell types individually upon LTβR stimulation, we determined that the stromal components of the spheroids produce proangiogenic molecules IL-6, IL-8, CXCL1 and CXCL5. This highlights the importance of stromal cells in tumor angiogenesis and is in line with mounting evidence describing the fundamental role that the tumor microenvironment plays in this process. Together these data support the hypothesis that noncanonical NF-κB signaling in tumor associated vasculature assists in tumor angiogenesis.

One of the benefits of the 3D model is that each cell type can be manipulated individually before incorporation into spheroids, making it possible to delineate cell specific mechanisms and contributions to angiogenesis. Here, we targeted NIK in ECs, as these are the cells expressing NIK in relevant patient tissues, and subsequently we found that NIK expression is critical for LTβR-induced angiogenesis associated with colorectal cancer, whereas it does not seem to affect GF-induced sprouting. This is in line with previous findings [23, 26]. Interestingly, in the context of LIGHT stimulation, the effect of siNIK is enhanced with the addition of bevacizumab, suggesting that LIGHT is to some extent linked to VEGF signaling. This has been described in a few cases in which VEGF was reported to up-regulate expression of LIGHT in macrophages [27] and alternatively, when tumors overexpressing LIGHT were xenografted onto mice, this led to an increase in VEGF in the tumors [28]. This may also explain our observation of increased VEGF transcripts in fibroblasts stimulated with LIGHT under hypoxia (Supplementary Figure 4). Additionally, this supports previous data indicating that LIGHT plays a role in angiogenesis as we have demonstrated that LIGHT stimulated vessel formation both in EC cultures and *ex vivo* murine aortic ring explants, and more recently, others showed that LIGHT-deficient mice exhibit reduced angiogenesis in a skin fibrosis model [23, 29].

Furthermore, our finding that targeting of NIK in EC can have a synergistic effect with bevacizumab in blocking vessel formation promoted by growth factors is remarkable. This has not been previously described and may have important implications for treatment strategies that focus on blocking classical activators of angiogenesis, such as VEGF, in addition to alternative facilitators, such as inflammatory molecules. Interestingly, a preclinical trial has already been conducted in which tumors resistant to anti-VEGF treatment xenografted onto mice were subsequently treated with a combination of a VEGF- and an IL-6 receptor inhibitor. This resulted in a total blockade of tumor growth, which was not achieved when either treatment was administered individually [30]. Particularly notable in that study was the enhanced production of IL-6, IL-8 and CXCL1 in the tumor lines resistant to anti-VEGF treatment, the same cytokines that were upregulated in stromal cells upon LTβR stimulation in our study. Consequently, targeting NIK or other components of the noncanonical NF-κB pathway in mice may yield similar results. The evidence presented here in conjunction with recent literature, suggests that further research into the contributions of the noncanonical NF-κB pathway to CRC angiogenesis are justified and may prove to be beneficial to improve treatment of the disease, particularly when combined with current therapeutic regimens that include bevacizumab.

## MATERIALS AND METHODS

### Patient material

Primary colorectal tumor tissues were received from the Department of Pathology of the Academic Medical Center (AMC). Specimens of liver metastasis of colorectal cancer were obtained during resection procedure from patients receiving no neoadjuvant treatment, chemotherapy alone or a combination of chemotherapy and bevacizumab prior to the operation. In patients receiving bevacizumab, as part of the preoperative systemic treatment, bevacizumab was withheld 6-8 weeks before surgery and the last cycle of chemotherapy was administered approximately 4 weeks before surgery. The present study was conducted in accordance with the Declaration of Helsinki and according to the guidelines of the ethics committee of the AMC. Detailed information on patient demographics and regimens of chemotherapy received before surgery are summarized in in Supplementary Table 1.

### Reagents

The following reagents were purchased from R&D systems (Rochester, MN, USA) and diluted to these final concentrations: LTβ (100 ng/mL), LIGHT (TNFSF14, 100 ng/mL), VEGF (10 ng/mL) and bFGF (10 ng/mL). Bevacizumab (Avastin, Basel, Switzerland) was a kind gift from the Pharmacy of the Academic Medical Center (Amsterdam, the Netherlands) and used at a final concentration of 125 µg/mL.

### Tissue immunohistochemistry

Healthy human colon, liver and CRC tissues were immunostained for NIK as previously described [23]. Liver metastasis tumor biopsies were formalin fixed, paraffin embedded and sectioned into 5μm slices and antigen retrieval completed using sodium citrate. CRC tissues were stained using antibodies against CD31 (JC70A DAKO, Glostrup, Denmark), SMA (M0851 DAKO, Glostrup, Denmark) and NIK (ab19144 Abcam, Cambridge, UK) in combination with secondary antibodies conjugated with alexa 647 (Invitrogen, Carlsbad, California, USA), alexa 568 (Invitrogen, Carlsbad, California, USA) or alexa 488 (Invitrogen, Carlsbad, California, USA). Subsequent imaging was performed at 40× magnification by confocal microscopy (Leica, Wetzlar, Germany).

### Cell culture

Primary human umbilical vein ECs (HUVEC) were cultured on gelatin-coated culture plates in Medium 199 (M199; Gibco, USA) supplemented with 0,1 μg/ml penicillin and streptomycin (pen/strep; Gibco, USA), 10 mM L-glutamine (Lonza, Switzerland), 0,025 U/ml heparin, 18,75 ng/ml EC growth serum (ECGS; Gibco, USA), and 20% fetal bovine serum (FBS; Biowest, France). When 80% confluent, cells were trypsinized (0.05% trypsin in phosphated buffered saline (PBS), Fresenius Kabi, Netherlands) and replated in a 1:3 density. Cells were used between passage 2 and 6. Normal human dermal fibroblasts (NHDF, Promocell GmbH, Heidelberg, Germany) were cultured in Dulbecco’s Modified Eagle’s Medium (DMEM; Gibco, USA) supplemented with 0,1 μg/ml pen/strep, 10 mM L-glutamine, 10 mM N-2-hydroxyethylpiperzine-N-2-ethane sulfonic acid (HEPES; Gibco, USA), 0.5 mg/ml Gentamicin (Gibco, USA), and 10% FBS. When 80–90% confluent, cells were trypsinized (0.05% trypsin in PBS) and replated in a 1:5 to 1:10 density. Colo320-HRS cells (ATCC, Manassas, USA) were cultured in Roswell Park Memorial Institute 1640 medium (RPMI; Gibco, USA) supplemented with 0,1 μg/ml pen/strep, 10 mM L-glutamine, and 10% FBS. When 80% confluent, cells were trypsinized (0.05% trypsin in PBS) and replated in a 1:10 density.

### 3D tumor stroma model

3D spheroid co-cultures were set up and immunostained using a similar approach as described previously [31], using EC Growth Medium-2 (EGM-2) (Lonza, Basel, Switzerland), 2% FCS (v/v), Hydrocortisone, Epithelial Growth Factor (EGF), Insulin-like Growth Factor-1 (IGF-1), ascorbic acid, GA-100, Heparin and bFGF/VEGF. Methocel solution was prepared by dissolving 6g methylcellulose in 500 ml M199 medium. Cells were incubated in 2 mM solution of CellTrackerTM green CMFDA, CellTrackerTM orange CMRA or CellTrackerTM deep red dye (Molecular probes, Invitrogen, UK). 750 HUVEC, 375 NHDF and 375 CRC (Colo320-HRS) cells were added to each well of 96 U-well suspension plate (Greiner BioOne, UK) in 150 µL of EGM-2 with 20% methocel(v/v). Cells formed spheroids overnight at 37° C. Afterwards, a solution of 1.5 mg/ml of rat-tail collagen type-I (BD Biosciences, UK) was prepared in EGM-2 medium and pH neutralized by 1 M NaOH. Initial layer was deposited in the center of wells of 4 well chamber slide (iBidi, Martinsried, Germany,) as droplets and set at 37° C. Spheroids were re-suspended in an equivalent solution of collagen type-I and deposited over first layer, and incubated at 37° C for 1h. After collagen gels set, 700 ul of RPMI medium containing 10% FCS, 100 ug/mL Pen/Strep, and 2 mM L-Glutamine including stimulants or inhibitors were added to wells and spheroids formed sprouts for 48hrs before fixation with 4% PFA (w/v) in HBSS (Invitrogen, Carlsbad, CA, USA). Sprouting was imaged by Leica TCS SP8-X confocal microscopy (Leica Camera AG, Wetzlar, Germany), using 10× magnification. Confocal images corresponding to pre-dyed EC sprouting were quantified using Leica QWin Plus (Leica Camera AG, Wetzlar, Germany) image analysis software. *O*f note: Different cancer cell lines grow at different rates and as such, varying ratios of CRC can be tested when trying to set up the model with other cell lines. Here we use HUVEC:NHDF:CRC at a ratio of 2:1:1 for the Colo320-HRS cell line.

### Gene expression

To analyze mRNA expression levels for VEGF, IL-6, IL-8, CXCL1 and CXCL5, cells were lysed and RNA was isolated using GenEluteTM Mammalian Total RNA Miniprep Kit (Sigma-Aldrich, Netherlands) according to protocol. cDNA was made using Thermofisher Strand cDNA synthesis kit (Thermofisher, USA). Subsequently, samples were plated in a 96 well plate containing sample cDNA, SybrGreen (Applied Biosystems, USA), and appropriate primers (VEGF; Fwd-ACTGCCATCCAATCGAGACC, Rev-GATCCGCATAATCTGCATGGTG, IL-6; Fwd-TGCAATAACCACCCCTGACC, Rev-ACTCCTTAAAGCTGCGCAGA, IL-8; Fwd-CAGGAATTGAATGGGTTTGC, Rev-AAACCAAGGCACAGTGGAAC, CXCL1; Fwd-GCTCCTGGTAGCCGCTG, Rev-TGTGGCTATGACTTCGGTTT, CXCL5; Fwd-AGACCACGCAAGGAGTTCAT, Rev-TCCTTGTTTCCACCGTCCAA). qPCR was performed and analyzed using StepOne Plus (Applied biosystems, USA).

### siRNA transfection

HUVECs were plated 250,000 cells per well in a 6-well plate and incubated overnight. Cells were transfected with either non-targeting or NIK-targeting siRNA at 50 nM final concentration (GE Dharmacon, Pittsburgh, PA, USA) using Dharmafect I (GE Dharmacon, Pittsburgh, PA, USA) as previously described [26]. Cells were collected 36 hours post-transfection and incorporated into spheroids. Confirmation of transcriptional knockdown of NIK is demonstrated in Supplementary Figure 8.

## SUPPLEMENTARY MATERIALS FIGURES AND TABLE


