# Supplementary material for: Silencing NIK potentiates anti-VEGF therapy in a novel 3D model of colorectal cancer angiogenesis
Source: Oncotarget. 2018 Jun 19;9(47):28445–55. doi: 10.18632/oncotarget.25442 (PMC6033358; doi:10.18632/oncotarget.25442)
Supplement: Supplementary file 1 [file oncotarget-09-28445-s001.pdf]

Silencing NIK potentiates anti-VEGF therapy in a novel 3D model of colorectal cancer angiogenesis

SUPPLEMENTARY MATERIALS

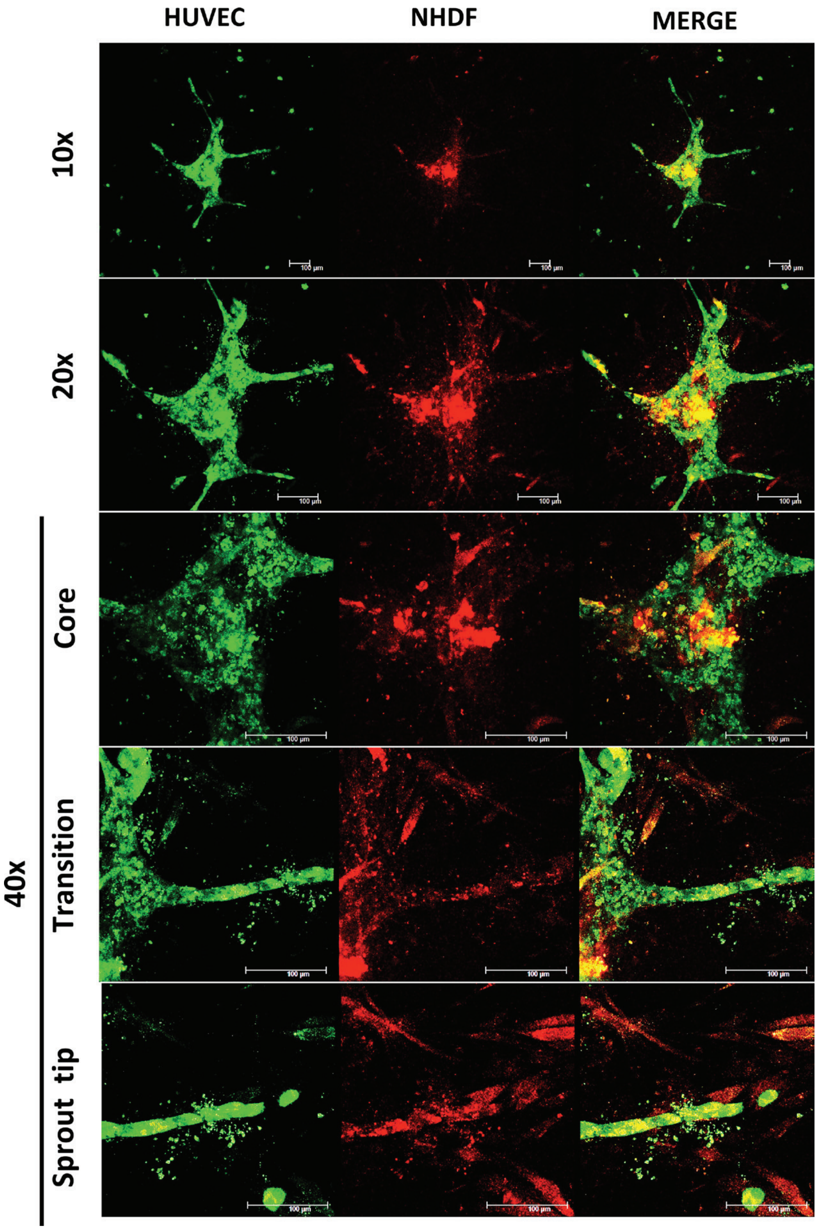

Supplementary Figure 1: Activation of the non-canonical NF-kappa B pathway induces angiogenesis in the 3D tumor-stroma model. Representative confocal images of a spheroid containing HUVEC, NHDF and Colo320 cells, stimulated with LT at 10×, 20× and 40× magnification. Depicted are the stromal component of a whole spheroid (top two panels) and details of the core, transition to sprout, or sprout tip (lower three panels).

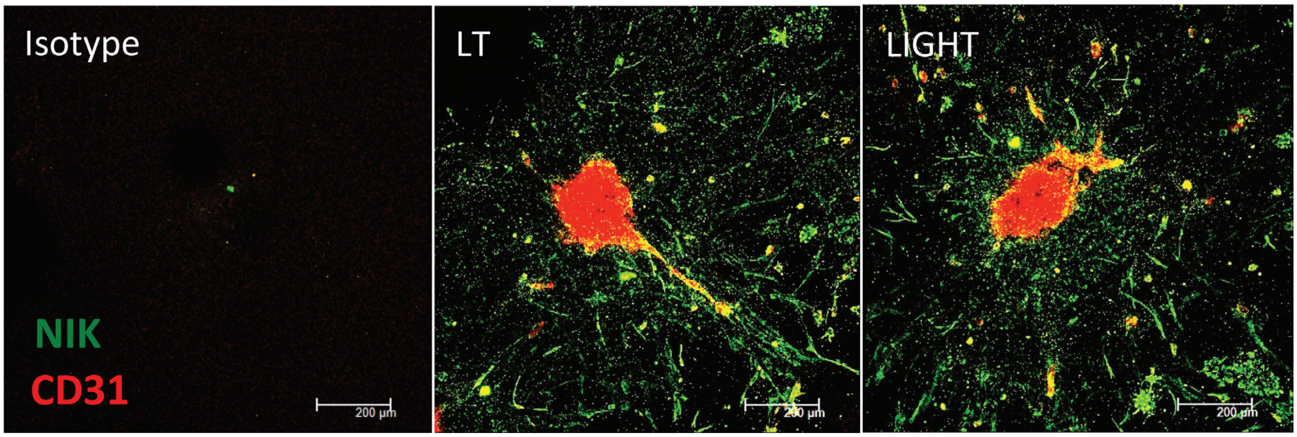

Supplementary Figure 2: Stimulation of lymphotoxin-beta receptor activates NIK in spheroid endothelial cells. Representative confocal images of a spheroid containing HUVEC, NHDF and Colo320 cells, stimulated with LT and LIGHT and subsequently immunostained for NIK and the endothelial cell marker, CD31. Images taken at 10× magnification.

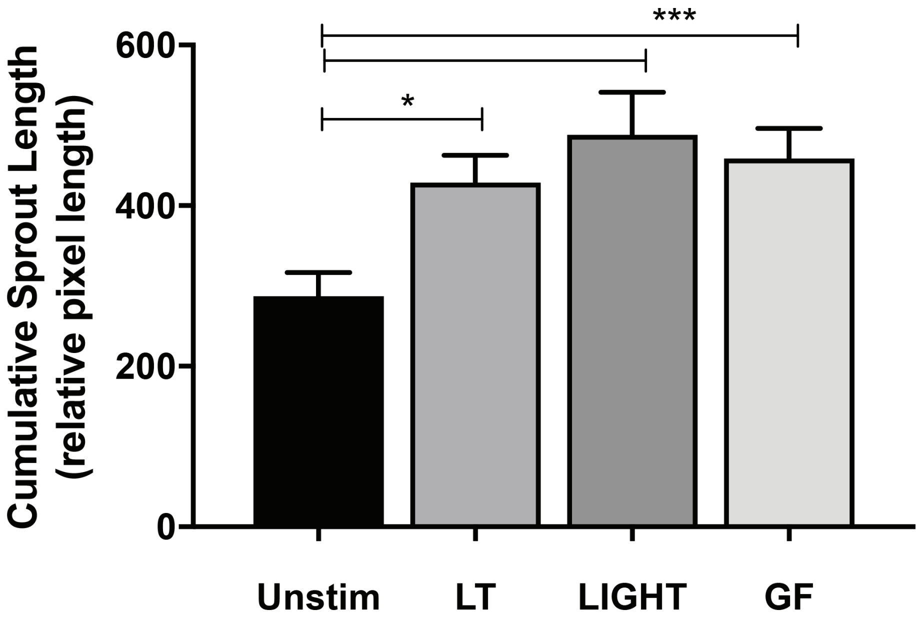

Supplementary Figure 3: Activation of the non-canonical pathway induces angiogenesis in the 3D tumor-stroma model under hypoxic conditions. Quantification of cumulative sprout length during hypoxia in spheroids unstimulated or stimulated with LT, LIGHT or GF. (n = 27–44 spheroids per condition). (*represents $p < 0.05$; ***represents $p < 0.001$).

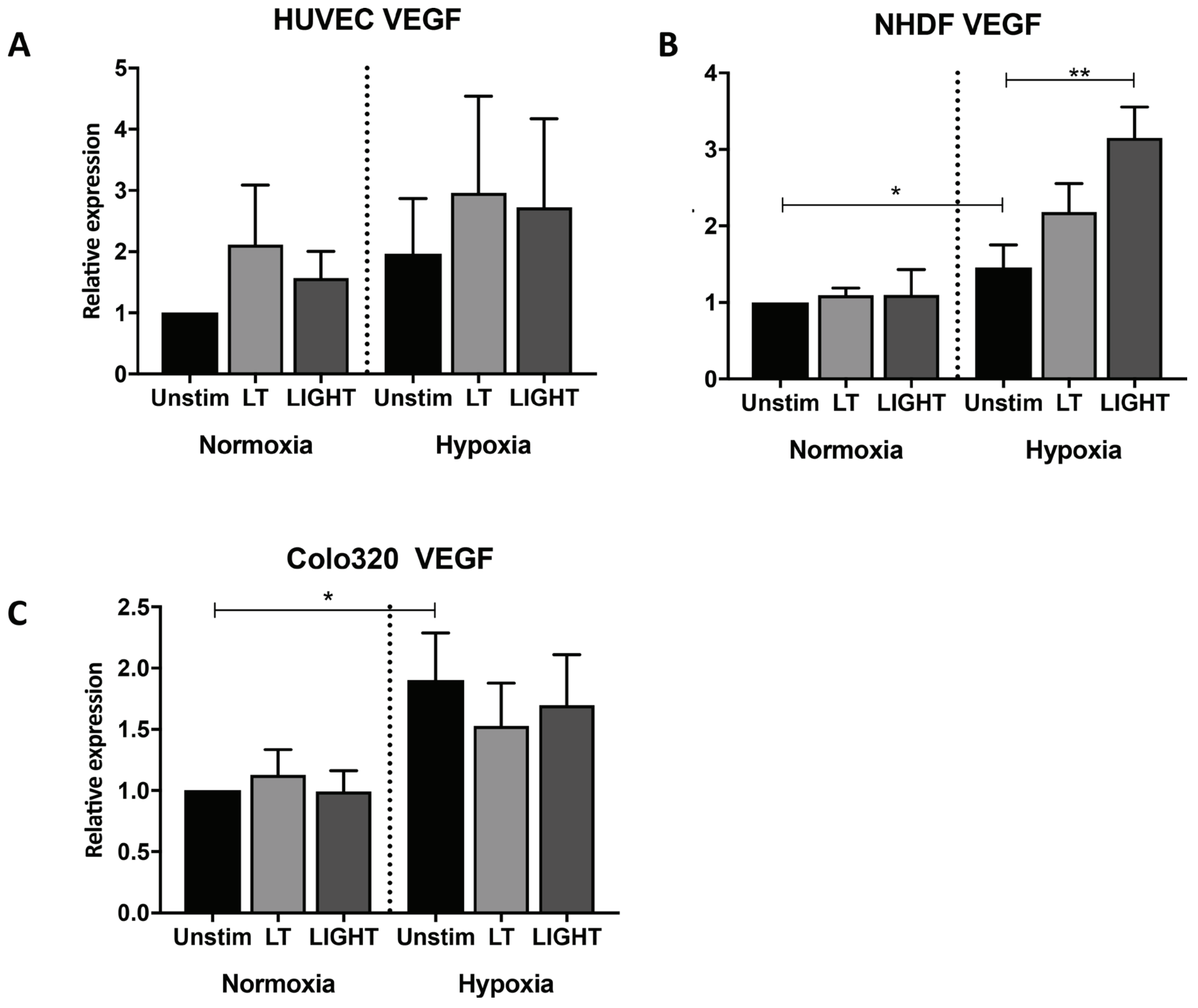

Supplementary Figure 4: Expression of VEGF in stromal and cancer cells upon LTβR activation. Gene expression levels of VEGF by real-time quantitative polymerase chain reaction following stimulation with LT or LIGHT in (**A**) Endothelial cells (HUVEC) (**B**) Fibroblasts (NHDF) and (**C**) Cancer cells (Colo320) under normoxic and hypoxic culture conditions (*n* = 6). (*signifies $p < 0.05$; **signifies $p < 0.01$).

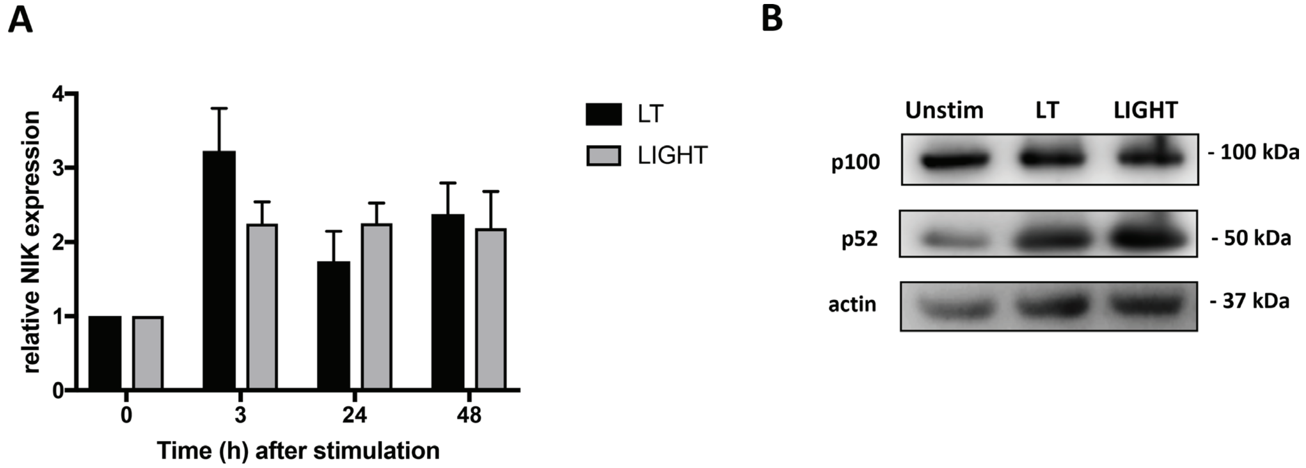

Supplementary Figure 5: Activation of the non-canonical NF-kappa B pathway in HUVEC upon LTβR activation. (**A**) HUVEC expression of NF-kappa B inducing kinase (NIK) upon stimulation with LT or LIGHT, by real-time quantitative polymerase chain reaction (*n* = 3 for LT, *n* = 4 for LIGHT). (**B**) Representative western blot image of p100 to p52 processing in HUVEC upon stimulation with LT or LIGHT (*n* = 4); actin was used as loading control.

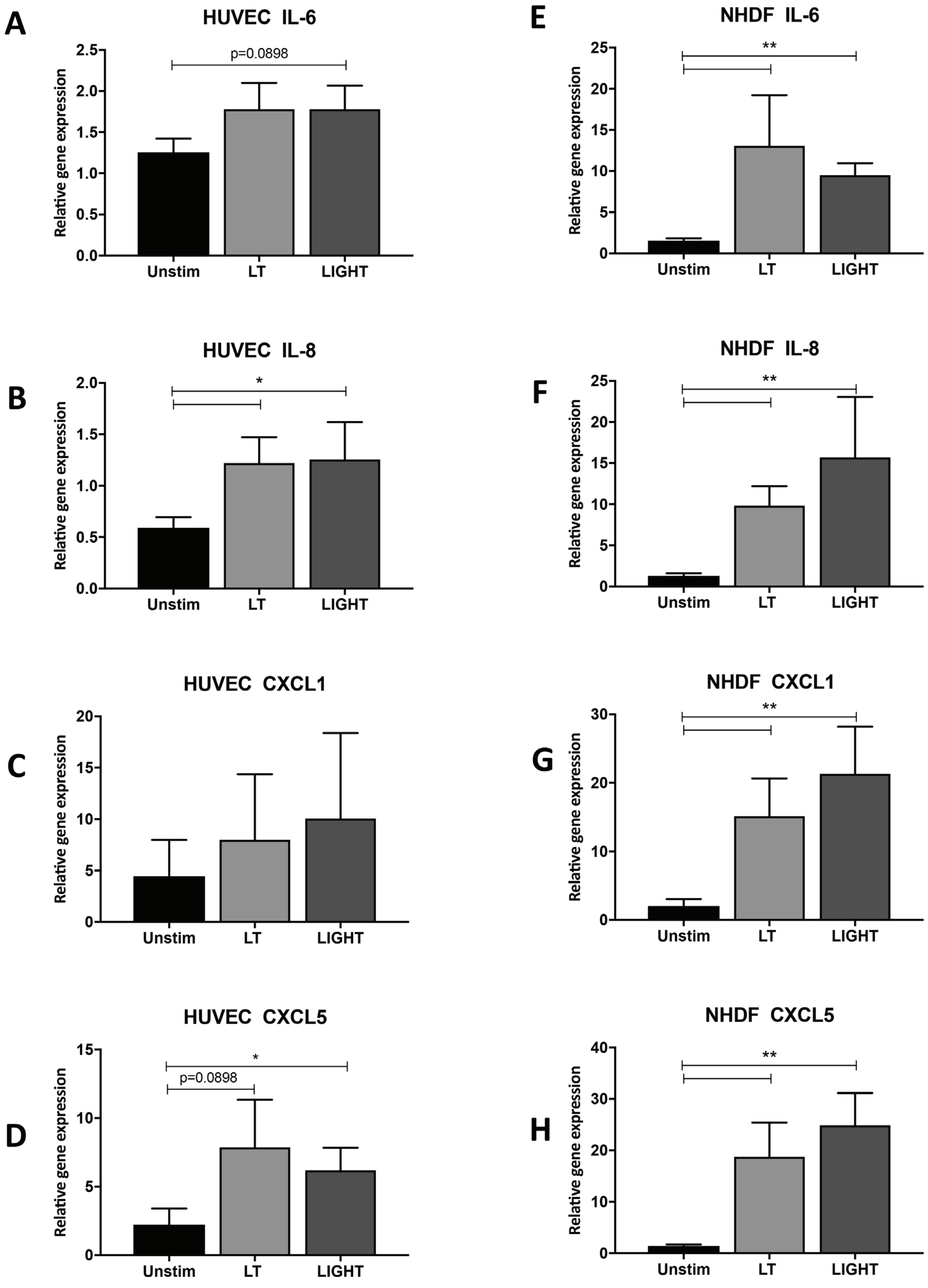

Supplementary Figure 6: Activation of the non-canonical NF-kappa B pathway induces expression of pro-angiogenic factors in stromal cells during hypoxia. HUVEC gene expression of (**A**) interleukin 6 (IL-6) (**B**) IL-8 (**C**) chemokine (C-X-C motif) ligand 1 (CXCL1) and (**D**) CXCL5, upon stimulation with LT or LIGHT during hypoxia, by real-time quantitative polymerase chain reaction ($n = 6$). Similarly, NHDF transcript levels of (**E**) IL-6, (**F**) IL-8, (**G**) CXCL1 and (**H**) CXCL5 ($n = 6$) following activation of the LTβR under hypoxic culture conditions. (*signifies $p < 0.05$; **signifies $p < 0.01$).

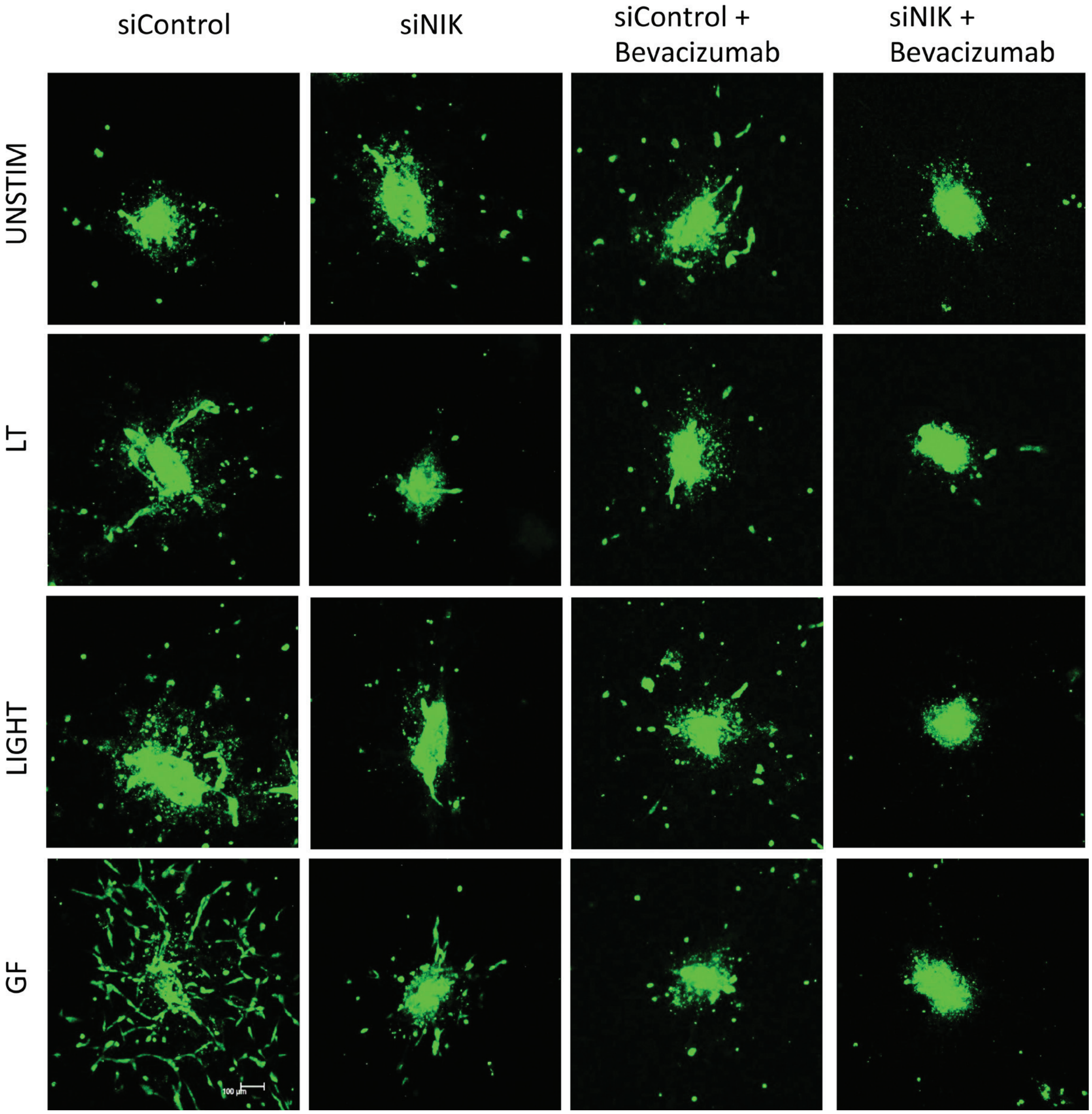

Supplementary Figure 7: Combined targeting of NIK in HUVEC and bevacizumab has a synergistic effect on blocking angiogenesis in the 3D tumor-stroma model. Representative confocal images of all conditions used for spheroid quantification. Spheroids contained HUVEC pretreated with nontargeting (siControl) or NIK targeting (siNIK) siRNA, NHDF and Colo320 cells, treated with or without bevacizumab (125 μg/mL), unstimulated or stimulated with LT, LIGHT or GF (*n* = 7–12 spheroids per condition). HUVEC are shown in green.

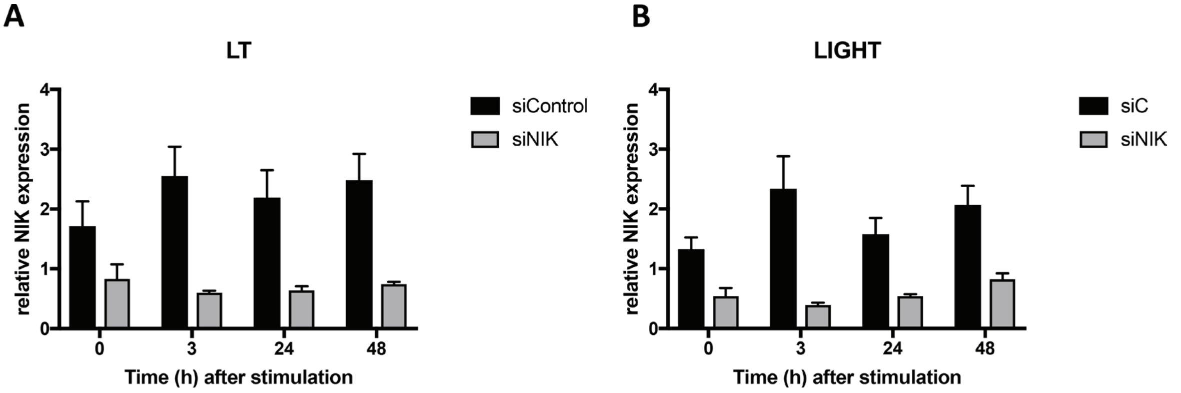

Supplementary Figure 8: Targeting NIK using siRNA reduces NIK expression in HUVEC upon LTβR activation. Expression of NIK in HUVEC treated with siNIK or siControl after stimulation with (**A**) LT or (**B**) LIGHT, by real-time quantitative polymerase chain reaction (n = 3 for LT, n = 4 for LIGHT).

Supplementary Table 1: Patient Characteristics

	**No neoadjuvant treatment (n = 13)**	**Chemotherapy Alone (n = 10)**	**Chemotherapy + Bevacizumab (n = 9)**
**Demographics:**			
Age, years	64 ± 8.49	58 ± 10.30	58 ± 10.09
Male (%)	10 (76.9%)	7 (70.0%)	7 (77.8%)
**Chemotherapy Regimen (%):**			
Capecitabine + Oxaliplatin	-	9 (90.0%)	4 (44.4%)
Capecitabine + Oxaliplatin followed by Capecitabine	-	-	1 (11.1%)
Capecitabine + Oxaliplatin followed by Capecitabine + Irinotecan	-	-	1 (11.1%)
Capecitabine + Oxaliplatin followed by 5-fluorouracil, Leukovorin, Irinotecan	-	-	1 (11.1%)
5-fluorouracil, Leucovorin, Oxaliplatin	-	-	1 (11.1%)
5-fluorouracil, Leukovorin, Irinotecan	-	-	1 (11.1%)
Capecitabine + Irinotecan	-	1 (10.0%)	-

Results are presented as mean ± standard deviation or numbers (percentages).
